# 10α-Hy­droxy-4,9-dimethyl-13-[(4-phenyl­piperazin-1-yl)meth­yl]-3,8,15-trioxatetra­cyclo­[10.3.0.0^2,4^.0^7,9^]tetra­decan-14-one

**DOI:** 10.1107/S1600536811042012

**Published:** 2011-10-22

**Authors:** Mohamed Moumou, Ahmed Benharref, Abdelghani Oudahmane, Ahmed Elhakmaoui, Moha Berraho

**Affiliations:** a Laboratoire de Chimie Biomoléculaire, Substances Naturelles et Réactivité, URAC 16, Faculté des Sciences Semlalia, BP 2390, Bd My Abdellah, 40000 Marrakech, Morocco; b Universite Blaise Pascal, Laboratoire des Mate’riaux Inorganiques, UMR CNRS 6002, 24 Avenue des Landais, 63177 Aubiere, France; c Laboratoire de Chimie Bioorganique et Analytique, URAC 22, BP 146, FSTM, Université Hassan II, Mohammedia-Casablanca 20810 Mohammedia, Morocco

## Abstract

The title compound, C_25_H_34_N_2_O_5_, was synthesized from 9α-hy­droxy­parthenolide (9α-hy­droxy-4,8-dimethyl-12-methyl­ene-3,14-dioxatricyclo­[9.3.0.0^2,4^]tetra­dec-7-en-13-one), which was isolated from the chloro­form extract of the aerial parts of *Anvillea radiata*. The mol­ecule contains a fused five- and ten-membered ring system. The ten-membered ring adopts an approximate chair–chair conformation, while the five-membered ring is in an envelope conformation, with the C atom closest to the hy­droxy group forming the flap. The piperazine ring is in a chair conformation. In the crystal, O—H⋯O hydrogen bonds connect mol­ecules into chains along [100]. Weak inter­molecular C—H⋯O hydrogen bonds are also present.

## Related literature

For background to the medicinal uses of the plant *Anvillea radiata*, see: Abdel Sattar *et al.* (1996 ▶); Bellakhdar (1997 ▶); El Hassany *et al.* (2004 ▶). For the reactivity of this sesquiterpene, see: Hwang *et al.* (2006 ▶); Neukirch *et al.* (2003 ▶); Neelakantan *et al.* (2009 ▶). For the synthesis, see: Moumou *et al.* (2010 ▶). For ring puckering parameters, see: Cremer & Pople (1975 ▶).
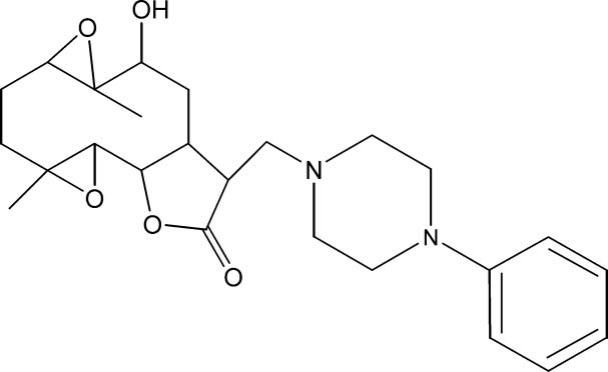


## Experimental

### 

#### Crystal data

C_25_H_34_N_2_O_5_
*M* *_r_* = 442.54
Orthorhombic, 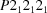

*a* = 7.7666 (5) Å
*b* = 9.6059 (8) Å
*c* = 31.181 (2) Å
*V* = 2326.2 (3) Å^3^
*Z* = 4
Mo *K*α radiation
μ = 0.09 mm^−1^
*T* = 298 K
0.45 × 0.36 × 0.28 mm

#### Data collection

Bruker X8 APEX CCD area-detector diffractometer
10922 measured reflections
2723 independent reflections
2362 reflections with *I* > 2σ(*I*)
*R*_int_ = 0.024

#### Refinement

*R*[*F* ^2^ > 2σ(*F* ^2^)] = 0.040
*wR*(*F* ^2^) = 0.104
*S* = 1.06
2723 reflections
293 parameters
H-atom parameters constrained
Δρ_max_ = 0.17 e Å^−3^
Δρ_min_ = −0.18 e Å^−3^

### 

Data collection: *APEX2* (Bruker, 2005 ▶); cell refinement: *SAINT* (Bruker, 2005 ▶); data reduction: *SAINT*; program(s) used to solve structure: *SHELXS97* (Sheldrick, 2008 ▶); program(s) used to refine structure: *SHELXS97* (Sheldrick, 2008 ▶); molecular graphics: *ORTEP-3 for Windows* (Farrugia,1997 ▶) and *PLATON* (Spek, 2009 ▶); software used to prepare material for publication: *WinGX* (Farrugia, 1999 ▶).

## Supplementary Material

Crystal structure: contains datablock(s) I, global. DOI: 10.1107/S1600536811042012/lh5350sup1.cif

Structure factors: contains datablock(s) I. DOI: 10.1107/S1600536811042012/lh5350Isup2.hkl

Additional supplementary materials:  crystallographic information; 3D view; checkCIF report

## Figures and Tables

**Table 1:** Hydrogen-bond geometry (Å, °)

*D*—H⋯*A*	*D*—H	H⋯*A*	*D*⋯*A*	*D*—H⋯*A*
O4—H4⋯O2^i^	0.82	2.11	2.902 (3)	161
C14—H14*B*⋯O5^ii^	0.96	2.59	3.289 (3)	129
C21—H21⋯O1^iii^	0.93	2.51	3.441 (4)	174

Symmetry codes: (i) 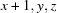; (ii) 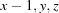; (iii) 
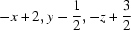
.

**Fig. 1.**
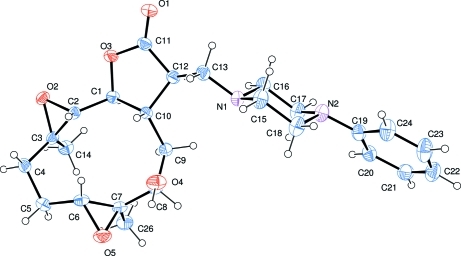
Molecular structure of the title compound with displacement ellipsoids drawn at the 30% probability level. H atoms are represented as small spheres of arbitrary radii.

**Fig. 2.**
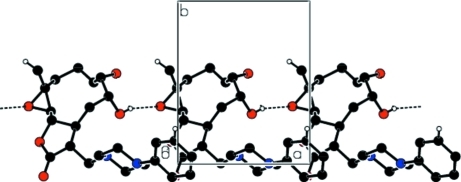
Packing view showing O–H···O hydrogen bonds as dashed lines. H atoms not involved in hydrogen bonding have been omitted for clarity.

## supplementary crystallographic information

### Comment

The natural sesquiterpene lactone, 9α - hydroxypartenolide is the main constituent of the chloroform extract of the aerial parts of *Anvillea radiata* (El Hassany *et al.*, 2004) and of *Anvillea garcini* (Abdel Sattar *et al.*, (1996). The reactivity of this sesquiterpene lactone and its derivatives has been the subject of several studies (Neukirch *et al.*, 2003; Hwang *et al.*, 2006; Neelakantan *et al.*, 2009), in order to prepare products of value which can be used in the pharmacological industry. In this context, we have synthesed from 9α-hydroxyparthenolide the 6β,7α- epoxy-9apha hydoxy partenolide (9α-hydroxy-4,8-dimethyl-12- methylen-3,14-dioxa-tricyclo[9.3.0.0^2^,^4^]tetradec-7-en-13-one) (Moumou *et al.*, 2010) and then prepared the title compound (I). The crystal structure of (I) is determined herein. The molecule contains a fused ring system and phenylpiperazine group as a substituent to a lactone ring. The molecular structure, Fig.1, shows that the lactone ring adopts an envelope conformation, as indicated by the Cremer & Pople (1975) puckering parameters Q = 0.347 (2)Å and φ = 75.6 (3)°. The ten-membered ring displays an approximate chair-chair conformation, while the piperazine ring has a perfect chair conformation with QT = 0.570 (2) Å, θ = 180.0 (2)° and φ2 = 150 (10)°. In the crystal structure, molecules are connected through O—H···O hydrogen bonds (Fig.2), forming chains along [100].

### Experimental

A mixture of 6β,7α-epoxy-9α-hydoxypartenolide (9α-hydroxy-4,8-dimethyl-12- methylen-3,14-dioxa-tricyclo[9.3.0.02,4]tetradec-7-en-13-one) (0.5 g, 2 mmol) and one equivalent of 1-phenylpiperazine in EtOH (20 ml) was stirred for twelve hours at room temperature. Then the reaction was stopped by adding water (10 ml) and extracted three times with ethyl acetate (3 *x* 20 ml). The combined organic layers were dried over anhydrous MgSO_4_, filtered and concentrated under vacuum to give 895 mg (1.8 mmol) of the title compound, which was recrystallized in ethyl acetate.

### Refinement

All H atoms were fixed geometrically and treated as riding with C—H = 0.96 Å (methyl), 0.97 Å (methylene), 0. 98Å (methine), O—H = 0.82Å and with *U*_iso_(H) = 1.2*U*_eq_ (methylene, methine) or *U*_iso_(H) = 1.5*U*_eq_ (methyl, OH). In the absence of significant anomalous scattering, the absolute configuration could not be reliably determined and thus the Friedel pairs were merged.

### Crystal data

**Table:**

C_25_H_34_N_2_O_5_	*F*(000) = 952
*M**_r_* = 442.54	*D*_x_ = 1.264 Mg m^−^^3^
Orthorhombic, *P*2_1_2_1_2_1_	Mo *K*α radiation, λ = 0.71073 Å
Hall symbol: P 2ac 2ab	Cell parameters from 10922 reflections
*a* = 7.7666 (5) Å	θ = 2.7–26.4°
*b* = 9.6059 (8) Å	µ = 0.09 mm^−^^1^
*c* = 31.181 (2) Å	*T* = 298 K
*V* = 2326.2 (3) Å^3^	Prism, colourless
*Z* = 4	0.45 × 0.36 × 0.28 mm

### Data collection

**Table:**

Bruker X8 APEX CCD area-detector diffractometer	2362 reflections with *I* > 2σ(*I*)
Radiation source: fine-focus sealed tube	*R*_int_ = 0.024
graphite	θ_max_ = 26.4°, θ_min_ = 2.7°
φ and ω scans	*h* = −9→9
10922 measured reflections	*k* = −11→7
2723 independent reflections	*l* = −38→34

### Refinement

**Table:**

Refinement on *F*^2^	Primary atom site location: structure-invariant direct methods
Least-squares matrix: full	Secondary atom site location: difference Fourier map
*R*[*F*^2^ > 2σ(*F*^2^)] = 0.040	Hydrogen site location: inferred from neighbouring sites
*wR*(*F*^2^) = 0.104	H-atom parameters constrained
*S* = 1.06	*w* = 1/[σ^2^(*F*_o_^2^) + (0.0537*P*)^2^ + 0.4378*P*] where *P* = (*F*_o_^2^ + 2*F*_c_^2^)/3
2723 reflections	(Δ/σ)_max_ < 0.001
293 parameters	Δρ_max_ = 0.17 e Å^−^^3^
0 restraints	Δρ_min_ = −0.18 e Å^−^^3^

### Special details

**Table:**

Geometry. All s.u.'s (except the s.u. in the dihedral angle between two l.s. planes) are estimated using the full covariance matrix. The cell s.u.'s are taken into account individually in the estimation of s.u.'s in distances, angles and torsion angles; correlations between s.u.'s in cell parameters are only used when they are defined by crystal symmetry. An approximate (isotropic) treatment of cell s.u.'s is used for estimating s.u.'s involving l.s. planes.

### Fractional atomic coordinates and isotropic or equivalent isotropic displacement parameters (Å^2^)

**Table:**

	*x*	*y*	*z*	*U*_iso_*/*U*_eq_	
C1	0.5441 (3)	0.2316 (2)	0.91768 (7)	0.0347 (5)	
H1	0.4879	0.1665	0.8979	0.042*	
C2	0.5370 (3)	0.1777 (2)	0.96267 (7)	0.0327 (5)	
H2	0.6143	0.2252	0.9827	0.039*	
C3	0.4930 (3)	0.0341 (3)	0.97477 (7)	0.0349 (5)	
C4	0.5705 (3)	−0.0184 (3)	1.01607 (8)	0.0415 (6)	
H4A	0.4920	−0.0840	1.0293	0.050*	
H4B	0.5859	0.0591	1.0356	0.050*	
C5	0.7438 (3)	−0.0892 (3)	1.00857 (8)	0.0435 (6)	
H5A	0.7981	−0.1076	1.0360	0.052*	
H5B	0.7252	−0.1777	0.9943	0.052*	
C6	0.8616 (3)	−0.0009 (3)	0.98170 (8)	0.0383 (6)	
H6	0.8588	0.0985	0.9888	0.046*	
C7	0.9154 (3)	−0.0312 (3)	0.93753 (8)	0.0383 (6)	
C8	0.9740 (3)	0.0848 (3)	0.90772 (8)	0.0404 (6)	
H8	1.0567	0.0437	0.8876	0.048*	
C9	0.8307 (4)	0.1514 (3)	0.88067 (8)	0.0416 (6)	
H9A	0.7518	0.0783	0.8720	0.050*	
H9B	0.8825	0.1885	0.8548	0.050*	
C10	0.7253 (3)	0.2680 (2)	0.90184 (7)	0.0311 (5)	
H10	0.7914	0.3042	0.9262	0.037*	
C11	0.5317 (3)	0.4573 (3)	0.89329 (8)	0.0414 (6)	
C12	0.6839 (3)	0.3904 (3)	0.87154 (7)	0.0376 (6)	
H12	0.6455	0.3523	0.8440	0.045*	
C13	0.8251 (4)	0.4953 (3)	0.86298 (8)	0.0431 (6)	
H13A	0.7729	0.5793	0.8516	0.052*	
H13B	0.8786	0.5194	0.8901	0.052*	
C14	0.4374 (4)	−0.0747 (3)	0.94329 (9)	0.0473 (6)	
H14A	0.3988	−0.0306	0.9174	0.071*	
H14B	0.3451	−0.1284	0.9554	0.071*	
H14C	0.5328	−0.1349	0.9369	0.071*	
C15	1.1040 (4)	0.5453 (3)	0.83687 (8)	0.0519 (7)	
H15A	1.1452	0.5467	0.8662	0.062*	
H15B	1.0668	0.6387	0.8295	0.062*	
C16	0.9013 (3)	0.4473 (3)	0.78904 (8)	0.0490 (7)	
H16A	0.8618	0.5393	0.7808	0.059*	
H16B	0.8051	0.3836	0.7864	0.059*	
C17	1.0437 (4)	0.4021 (3)	0.75924 (8)	0.0509 (7)	
H17A	1.0794	0.3082	0.7664	0.061*	
H17B	1.0018	0.4018	0.7299	0.061*	
C18	1.2483 (4)	0.5027 (4)	0.80761 (8)	0.0542 (8)	
H18A	1.3418	0.5691	0.8100	0.065*	
H18B	1.2915	0.4122	0.8163	0.065*	
C19	1.3260 (4)	0.4797 (3)	0.73301 (8)	0.0448 (6)	
C20	1.3357 (4)	0.3688 (3)	0.70444 (8)	0.0473 (6)	
H20	1.2490	0.3020	0.7040	0.057*	
C21	1.4749 (4)	0.3575 (4)	0.67644 (8)	0.0571 (8)	
H21	1.4797	0.2835	0.6573	0.069*	
C22	1.6044 (4)	0.4538 (4)	0.67685 (9)	0.0645 (9)	
H22	1.6992	0.4434	0.6589	0.077*	
C23	1.5932 (4)	0.5657 (4)	0.70384 (10)	0.0714 (10)	
H23	1.6792	0.6331	0.7035	0.086*	
C24	1.4562 (4)	0.5797 (4)	0.73158 (9)	0.0615 (8)	
H24	1.4503	0.6568	0.7496	0.074*	
C26	0.8708 (4)	−0.1630 (3)	0.91409 (9)	0.0535 (7)	
H26A	0.8222	−0.2288	0.9338	0.080*	
H26B	0.9730	−0.2015	0.9015	0.080*	
H26C	0.7886	−0.1428	0.8919	0.080*	
N1	0.9589 (3)	0.4506 (2)	0.83356 (6)	0.0398 (5)	
N2	1.1901 (3)	0.4959 (2)	0.76275 (6)	0.0461 (6)	
O1	0.4819 (3)	0.5750 (2)	0.89055 (6)	0.0565 (5)	
O2	0.3686 (2)	0.14622 (18)	0.97984 (5)	0.0400 (4)	
O3	0.4524 (2)	0.36458 (19)	0.91853 (5)	0.0447 (4)	
O5	1.0321 (2)	−0.0547 (2)	0.97319 (6)	0.0543 (5)	
O4	1.0600 (2)	0.19268 (19)	0.92937 (7)	0.0538 (5)	
H4	1.1392	0.1600	0.9436	0.081*	

### Atomic displacement parameters (Å^2^)

**Table:**

	*U*^11^	*U*^22^	*U*^33^	*U*^12^	*U*^13^	*U*^23^
C1	0.0319 (12)	0.0320 (12)	0.0403 (12)	−0.0020 (11)	−0.0021 (11)	0.0019 (10)
C2	0.0281 (11)	0.0336 (12)	0.0364 (11)	−0.0008 (11)	0.0030 (10)	0.0008 (9)
C3	0.0274 (11)	0.0351 (12)	0.0421 (12)	−0.0020 (10)	0.0068 (10)	0.0002 (10)
C4	0.0464 (14)	0.0380 (13)	0.0402 (12)	−0.0040 (12)	0.0048 (11)	0.0072 (11)
C5	0.0486 (14)	0.0374 (14)	0.0446 (13)	0.0030 (12)	−0.0051 (12)	0.0065 (11)
C6	0.0304 (12)	0.0343 (13)	0.0502 (13)	0.0040 (11)	−0.0070 (11)	−0.0018 (11)
C7	0.0330 (12)	0.0338 (13)	0.0479 (13)	0.0062 (11)	−0.0035 (11)	−0.0007 (11)
C8	0.0326 (12)	0.0363 (13)	0.0522 (14)	0.0066 (11)	0.0058 (11)	−0.0029 (11)
C9	0.0485 (15)	0.0362 (13)	0.0401 (12)	0.0049 (13)	0.0062 (12)	0.0012 (11)
C10	0.0303 (12)	0.0302 (12)	0.0328 (11)	−0.0005 (10)	−0.0014 (9)	0.0020 (9)
C11	0.0404 (13)	0.0420 (14)	0.0417 (13)	0.0051 (13)	−0.0057 (11)	0.0079 (11)
C12	0.0414 (13)	0.0336 (13)	0.0377 (12)	0.0019 (11)	−0.0008 (11)	0.0024 (10)
C13	0.0503 (14)	0.0352 (13)	0.0438 (13)	−0.0031 (13)	0.0073 (12)	0.0012 (11)
C14	0.0444 (14)	0.0394 (15)	0.0582 (15)	−0.0041 (13)	0.0002 (13)	−0.0033 (12)
C15	0.0527 (16)	0.0597 (18)	0.0434 (13)	−0.0120 (16)	0.0041 (12)	−0.0116 (13)
C16	0.0419 (14)	0.0639 (18)	0.0413 (13)	−0.0058 (15)	−0.0014 (11)	0.0009 (13)
C17	0.0485 (15)	0.0646 (18)	0.0396 (13)	−0.0098 (16)	−0.0015 (12)	−0.0086 (13)
C18	0.0467 (14)	0.073 (2)	0.0431 (13)	−0.0101 (15)	0.0003 (12)	−0.0112 (15)
C19	0.0479 (14)	0.0498 (15)	0.0368 (12)	−0.0031 (14)	0.0010 (11)	0.0005 (12)
C20	0.0538 (16)	0.0476 (15)	0.0403 (13)	−0.0030 (15)	0.0013 (13)	0.0019 (12)
C21	0.069 (2)	0.0614 (18)	0.0413 (14)	0.0143 (19)	0.0050 (14)	0.0000 (14)
C22	0.0538 (18)	0.098 (3)	0.0419 (15)	0.003 (2)	0.0075 (13)	0.0051 (17)
C23	0.0574 (19)	0.099 (3)	0.0578 (18)	−0.030 (2)	0.0051 (16)	0.0013 (19)
C24	0.0633 (18)	0.068 (2)	0.0528 (16)	−0.0199 (19)	0.0080 (15)	−0.0106 (15)
C26	0.0663 (19)	0.0335 (14)	0.0608 (16)	0.0041 (14)	0.0044 (15)	−0.0079 (13)
N1	0.0409 (11)	0.0424 (12)	0.0362 (10)	−0.0028 (11)	0.0019 (9)	−0.0001 (9)
N2	0.0454 (12)	0.0543 (14)	0.0386 (10)	−0.0091 (12)	0.0029 (10)	−0.0090 (10)
O1	0.0590 (12)	0.0477 (11)	0.0627 (11)	0.0188 (11)	0.0078 (10)	0.0146 (9)
O2	0.0310 (8)	0.0414 (9)	0.0477 (9)	0.0018 (8)	0.0081 (7)	0.0000 (8)
O3	0.0333 (9)	0.0458 (10)	0.0548 (10)	0.0089 (9)	0.0045 (8)	0.0136 (9)
O5	0.0371 (10)	0.0594 (12)	0.0662 (12)	0.0123 (10)	−0.0069 (9)	0.0060 (10)
O4	0.0350 (10)	0.0429 (11)	0.0834 (14)	−0.0036 (9)	−0.0090 (10)	0.0003 (10)

### Geometric parameters (Å, °)

**Table:**

C1—O3	1.463 (3)	C13—H13A	0.9700
C1—C2	1.496 (3)	C13—H13B	0.9700
C1—C10	1.532 (3)	C14—H14A	0.9600
C1—H1	0.9800	C14—H14B	0.9600
C2—O2	1.445 (3)	C14—H14C	0.9600
C2—C3	1.471 (3)	C15—N1	1.452 (3)
C2—H2	0.9800	C15—C18	1.502 (4)
C3—O2	1.456 (3)	C15—H15A	0.9700
C3—C14	1.497 (4)	C15—H15B	0.9700
C3—C4	1.508 (3)	C16—N1	1.459 (3)
C4—C5	1.526 (4)	C16—C17	1.508 (4)
C4—H4A	0.9700	C16—H16A	0.9700
C4—H4B	0.9700	C16—H16B	0.9700
C5—C6	1.503 (4)	C17—N2	1.455 (3)
C5—H5A	0.9700	C17—H17A	0.9700
C5—H5B	0.9700	C17—H17B	0.9700
C6—O5	1.446 (3)	C18—N2	1.471 (3)
C6—C7	1.468 (3)	C18—H18A	0.9700
C6—H6	0.9800	C18—H18B	0.9700
C7—O5	1.452 (3)	C19—C20	1.390 (4)
C7—C26	1.502 (4)	C19—C24	1.395 (4)
C7—C8	1.521 (4)	C19—N2	1.414 (3)
C8—O4	1.406 (3)	C20—C21	1.394 (4)
C8—C9	1.536 (3)	C20—H20	0.9300
C8—H8	0.9800	C21—C22	1.367 (5)
C9—C10	1.537 (3)	C21—H21	0.9300
C9—H9A	0.9700	C22—C23	1.368 (5)
C9—H9B	0.9700	C22—H22	0.9300
C10—C12	1.542 (3)	C23—C24	1.378 (4)
C10—H10	0.9800	C23—H23	0.9300
C11—O1	1.198 (3)	C24—H24	0.9300
C11—O3	1.339 (3)	C26—H26A	0.9600
C11—C12	1.507 (3)	C26—H26B	0.9600
C12—C13	1.513 (4)	C26—H26C	0.9600
C12—H12	0.9800	O4—H4	0.8200
C13—N1	1.451 (3)		
			
O3—C1—C2	105.50 (18)	N1—C13—H13A	108.4
O3—C1—C10	104.70 (18)	C12—C13—H13A	108.4
C2—C1—C10	114.50 (19)	N1—C13—H13B	108.4
O3—C1—H1	110.6	C12—C13—H13B	108.4
C2—C1—H1	110.6	H13A—C13—H13B	107.4
C10—C1—H1	110.6	C3—C14—H14A	109.5
O2—C2—C3	59.88 (14)	C3—C14—H14B	109.5
O2—C2—C1	116.93 (19)	H14A—C14—H14B	109.5
C3—C2—C1	125.0 (2)	C3—C14—H14C	109.5
O2—C2—H2	114.5	H14A—C14—H14C	109.5
C3—C2—H2	114.5	H14B—C14—H14C	109.5
C1—C2—H2	114.5	N1—C15—C18	111.4 (2)
O2—C3—C2	59.19 (15)	N1—C15—H15A	109.3
O2—C3—C14	113.4 (2)	C18—C15—H15A	109.3
C2—C3—C14	123.6 (2)	N1—C15—H15B	109.3
O2—C3—C4	114.79 (19)	C18—C15—H15B	109.3
C2—C3—C4	116.1 (2)	H15A—C15—H15B	108.0
C14—C3—C4	116.2 (2)	N1—C16—C17	111.6 (2)
C3—C4—C5	111.7 (2)	N1—C16—H16A	109.3
C3—C4—H4A	109.3	C17—C16—H16A	109.3
C5—C4—H4A	109.3	N1—C16—H16B	109.3
C3—C4—H4B	109.3	C17—C16—H16B	109.3
C5—C4—H4B	109.3	H16A—C16—H16B	108.0
H4A—C4—H4B	107.9	N2—C17—C16	110.4 (2)
C6—C5—C4	111.8 (2)	N2—C17—H17A	109.6
C6—C5—H5A	109.3	C16—C17—H17A	109.6
C4—C5—H5A	109.3	N2—C17—H17B	109.6
C6—C5—H5B	109.3	C16—C17—H17B	109.6
C4—C5—H5B	109.3	H17A—C17—H17B	108.1
H5A—C5—H5B	107.9	N2—C18—C15	111.1 (2)
O5—C6—C7	59.77 (15)	N2—C18—H18A	109.4
O5—C6—C5	117.3 (2)	C15—C18—H18A	109.4
C7—C6—C5	125.7 (2)	N2—C18—H18B	109.4
O5—C6—H6	114.2	C15—C18—H18B	109.4
C7—C6—H6	114.2	H18A—C18—H18B	108.0
C5—C6—H6	114.2	C20—C19—C24	117.9 (3)
O5—C7—C6	59.36 (15)	C20—C19—N2	123.0 (2)
O5—C7—C26	112.7 (2)	C24—C19—N2	119.1 (2)
C6—C7—C26	123.9 (2)	C19—C20—C21	120.2 (3)
O5—C7—C8	113.3 (2)	C19—C20—H20	119.9
C6—C7—C8	120.8 (2)	C21—C20—H20	119.9
C26—C7—C8	112.9 (2)	C22—C21—C20	120.8 (3)
O4—C8—C7	112.9 (2)	C22—C21—H21	119.6
O4—C8—C9	107.5 (2)	C20—C21—H21	119.6
C7—C8—C9	115.1 (2)	C21—C22—C23	119.4 (3)
O4—C8—H8	107.0	C21—C22—H22	120.3
C7—C8—H8	107.0	C23—C22—H22	120.3
C9—C8—H8	107.0	C22—C23—C24	120.8 (3)
C8—C9—C10	117.0 (2)	C22—C23—H23	119.6
C8—C9—H9A	108.0	C24—C23—H23	119.6
C10—C9—H9A	108.0	C23—C24—C19	120.8 (3)
C8—C9—H9B	108.0	C23—C24—H24	119.6
C10—C9—H9B	108.0	C19—C24—H24	119.6
H9A—C9—H9B	107.3	C7—C26—H26A	109.5
C1—C10—C9	117.5 (2)	C7—C26—H26B	109.5
C1—C10—C12	100.36 (18)	H26A—C26—H26B	109.5
C9—C10—C12	113.83 (19)	C7—C26—H26C	109.5
C1—C10—H10	108.2	H26A—C26—H26C	109.5
C9—C10—H10	108.2	H26B—C26—H26C	109.5
C12—C10—H10	108.2	C13—N1—C15	108.97 (19)
O1—C11—O3	121.5 (2)	C13—N1—C16	112.8 (2)
O1—C11—C12	128.6 (2)	C15—N1—C16	108.6 (2)
O3—C11—C12	109.9 (2)	C19—N2—C17	117.8 (2)
C11—C12—C13	111.3 (2)	C19—N2—C18	113.5 (2)
C11—C12—C10	102.31 (19)	C17—N2—C18	109.8 (2)
C13—C12—C10	117.7 (2)	C2—O2—C3	60.93 (15)
C11—C12—H12	108.4	C11—O3—C1	110.30 (18)
C13—C12—H12	108.4	C6—O5—C7	60.87 (15)
C10—C12—H12	108.4	C8—O4—H4	109.5
N1—C13—C12	115.7 (2)		

### Hydrogen-bond geometry (Å, °)

**Table:**

*D*—H···*A*	*D*—H	H···*A*	*D*···*A*	*D*—H···*A*
O4—H4···O2^i^	0.82	2.11	2.902 (3)	161
C14—H14B···O5^ii^	0.96	2.59	3.289 (3)	129
C21—H21···O1^iii^	0.93	2.51	3.441 (4)	174

Symmetry codes: (i) *x*+1, *y*, *z*; (ii) *x*−1, *y*, *z*; (iii) −*x*+2, *y*−1/2, −*z*+3/2.
